# Prediction of intensive care admission and hospital mortality in COVID-19 patients using demographics and baseline laboratory data

**DOI:** 10.1016/j.clinsp.2023.100183

**Published:** 2023-03-10

**Authors:** Vivian I. Avelino-Silva, Thiago J. Avelino-Silva, Marlon J.R. Aliberti, Juliana C. Ferreira, Vilson Cobello Junior, Katia R. Silva, Jose E. Pompeu, Leila Antonangelo, Marcello M. Magri, Tarcisio E.P. Barros Filho, Heraldo P. Souza, Esper G. Kallás, Study Group

**Affiliations:** a Department of Infectious and Parasitic Diseases, Faculdade de Medicina da Universidade de São Paulo, SP, Brazil; b Laboratório de Investigação Médica em Envelhecimento (LIM-66), Serviço de Geriatria, Hospital das Clínicas HCFMUSP, Faculdade de Medicina, Universidade de São Paulo, SP, Brazil; c Divisão de Pneumologia, Instituto do Coração, Hospital das Clínicas HCFMUSP, Faculdade de Medicina, Universidade de São Paulo, SP, Brazil; d Núcleo Especializado em Tecnologia da Informação, Hospital das Clínicas HCFMUSP, Faculdade de Medicina, Universidade de São Paulo, SP, Brazil; e Instituto do Coração, Hospital das Clínicas HCFMUSP, Faculdade de Medicina, Universidade de São Paulo, SP, Brazil; f Departamento de Fisioterapia, Fonoaudiologia e Terapia Ocupacional, Faculdade de Medicina, Universidade de São Paulo, SP, Brazil; g Laboratório Central, Hospital das Clínicas HCFMUSP, Faculdade de Medicina, Universidade de São Paulo, SP, Brazil; h Instituto de Ortopedia e Traumatologia, Hospital das Clínicas HCFMUSP, Faculdade de Medicina, Universidade de São Paulo, SP, Brazil; i Emergency Department, Hospital das Clínicas HCFMUSP, Faculdade de Medicina, Universidade de São Paulo, SP, Brazil

**Keywords:** COVID-19, SARS-CoV-2, Critical Care, Mortality, Intensive Care, Risk Score, Prediction

## Abstract

**Introduction:**

Optimized allocation of medical resources to patients with COVID-19 has been a critical concern since the onset of the pandemic.

**Methods:**

In this retrospective cohort study, the authors used data from a Brazilian tertiary university hospital to explore predictors of Intensive Care Unit (ICU) admission and hospital mortality in patients admitted for COVID-19. Our primary aim was to create and validate prediction scores for use in hospitals and emergency departments to aid clinical decisions and resource allocation.

**Results:**

The study cohort included 3,022 participants, of whom 2,485 were admitted to the ICU; 1968 survived, and 1054 died in the hospital. From the complete cohort, 1,496 patients were randomly assigned to the derivation sample and 1,526 to the validation sample. The final scores included age, comorbidities, and baseline laboratory data. The areas under the receiver operating characteristic curves were very similar for the derivation and validation samples. Scores for ICU admission had a 75% accuracy in the validation sample, whereas scores for death had a 77% accuracy in the validation sample. The authors found that including baseline flu-like symptoms in the scores added no significant benefit to their accuracy. Furthermore, our scores were more accurate than the previously published NEWS-2 and 4C Mortality Scores.

**Discussion and conclusions:**

The authors developed and validated prognostic scores that use readily available clinical and laboratory information to predict ICU admission and mortality in COVID-19. These scores can become valuable tools to support clinical decisions and improve the allocation of limited health resources.

## Highlights

• Prediction scores can be used to support clinical decisions and resource allocation.
• The authors used data from 3,022 hospitalized patients with COVID-19, of whom 1054 died.
• The final scores included age, comorbidities, and baseline laboratory data.
• Accuracy was 75% for ICU admission and 77% for death in the validation sample.
• Our scores were more accurate than the previous NEWS-2 and 4C Mortality Scores.

## Introduction

In the three years since the first cases of COVID-19 were identified in China, more than 676 million people have been diagnosed with COVID-19 worldwide, and more than 6.8 million have died from its complications. Notably, some countries held higher burdens of cases and deaths, including the United States, India, and Brazil.^1^ Data on the overall life expectancy and years of life lost show that most nations with reliable mortality data witnessed substantial reductions in life expectancy, with more than 28 million excess years of life lost in 2020 in 31 countries.^2^

A severe shortage of medical resources was reported during the peak phase of COVID-19 infections and hospitalizations in several regions. The scarcity of crucial resources such as Intensive Care Units (ICU) beds, mechanical ventilation devices, and protective gear for healthcare workers, as well as limited supplies of sedative medications, frequently resulted in inadequate protection for healthcare staff, reduced patient admissions, and restricted access to medical care. Although these issues challenged even some of the world's most affluent countries,^3^, ^4^, ^5^, ^6^ the impact of COVID-19 and other highly transmissible diseases is undeniably more dramatic in middle- and lower-income nations, where access to medical resources is limited even in usual conditions.^7^^,^^8^ In Brazil, there was a significant disparity in healthcare access, resulting in a high mortality rate among ICU patients that ranged from 13% to 57%.^9^, ^10^, ^11^ More critical examples were observed in Colombia,^12^ India,^13^ and Manaus, the capital of Amazonas state in Brazil, where hospitals ran out of oxygen supplies during a surge of SARS-CoV-2 infections in January 2021.^14^

Strategies to identify patients for whom scarce treatment and support interventions should be prioritized are crucial in this context.^15^ With the rising rates of COVID-19 vaccination in several countries^16^ it is less likely that the number of severe COVID-19 cases will reach the levels seen in 2020 and 2021. However, the emergence of new virus variants with potentially higher transmissibility and virulence,^17^ and the slow pace of vaccination coverage in several places^16^ could still result in severe stress for healthcare services.

In this study, the authors used data from a large Brazilian tertiary university hospital to explore predictors of ICU admission and hospital mortality in patients admitted for COVID-19 and to develop and validate prediction models that might be used as clinical decision tools for resource allocation in day-to-day emergency care.

## Methods

In this retrospective cohort study, the authors used Electronic Health Records (EHR) from COVID-19-related admissions to the largest referral hospital for the disease in Sao Paulo, Brazil. The authors developed a prediction score for intensive care admission and hospital mortality using demographics and baseline clinical variables.

Hospital das Clinicas, University of Sao Paulo Medical School (HCFMUSP), is a renowned 2,200-bed teaching hospital complex that specializes in providing high-level medical and surgical care. Between March 2020 and September 2020, its 900-bed central building was designated by the Sao Paulo State's Health Department to operate as a special COVID-19 treatment center, receiving SARS-CoV-2-infected patients from 278 secondary hospitals located in 85 cities, mainly in the Sao Paulo metropolitan area. Additionally, its intensive care capacity was increased four-fold with the conversion of regular wards to ICUs, totaling 300 ICU beds. Throughout the pandemic, COVID-19 care followed institutional protocols in our hospital.

### Participants and data collection

The authors analyzed data from consecutive patients (> 14 years) diagnosed with COVID-19 who were admitted as inpatients for at least 24 hours between March and August 2020. The presence of SARS-CoV-2 infection was confirmed through either RT-PCR or serology testing. In instances where RT-PCR testing was not conducted within ten days of symptom onset, serology was utilized as a confirmatory test for probable COVID-19 cases. The authors excluded patients with nosocomial COVID-19 infection, defined as patients admitted to the hospital for other causes who were infected with SARS-Cov-2 during their hospitalization.

The authors extracted data on the following variables: demographics; comorbidities; COVID-19 symptoms on admission; baseline laboratory tests; ICU admission; need for mechanical ventilation; severity of disease at ICU admission measured with Simplified Acute Physiology Score 3 (SAPS-3); and clinical outcomes, including death, discharge, or referral to another healthcare facility. Data from each participant were collected from EHR and compiled by a trained research team using standardized web-based forms and Research Electronic Data Capture (REDCap)^18^ resources.

### Data analysis

Numeric variables were reported as means and Standard Deviations (SDs) or medians and Interquartile Ranges (IQR), according to their distribution. Occasionally, variables were also stratified into categories to simplify their clinical interpretation. Categorical variables were reported as counts and proportions. The authors then used demographic, clinical, and laboratory data to develop prediction scoring systems.

The authors randomly split our participants into derivation and validation samples using a 1:1 ratio and selected 25 variables to feed our models based on their clinical relevance and causal relations: (1) Demographics: age, sex, race/ethnicity; (2) Clinical history: hypertension, diabetes mellitus, heart disease, stroke history, chronic obstructive pulmonary disease, rheumatologic disease, cancer; (3) COVID-19 symptoms: fever, muscle pain, dyspnea, cough, dysgeusia or anosmia, headache, diarrhea; (4) Admission laboratory: hemoglobin, neutrophile-to-lymphocyte ratio, creatinine, C-reactive protein. The model including the complete list of independent variables for each outcome was defined as Model 1. As sensitivity analyses, the authors also examined our models excluding the reported COVID-19 symptoms, as these variables were more likely to be affected by information bias, particularly among patients with a more severe clinical presentation on admission. The model excluding COVID-19 symptoms for each outcome was defined as Model 2.

Subsequently, the authors explored the association between each variable of interest and the primary outcomes in univariable logistic regressions and used stepwise logistic regression models to select the final predictors to build our scoring system (variables with p-values < 0.1 were retained). The authors used variation inflation factors to assess for collinearity.

In accordance with the resulting models, the authors attributed points to each predictor dividing their respective beta coefficients by the lowest available beta coefficient and rounding the results to the nearest integer (0 or 5). The authors then used the sum of these points to estimate risk scores for our sample and examine their accuracy to predict hospital death and ICU admission. The authors validated the performances of the risk scoring systems using Receiver Operating Characteristic (ROC) analyses and test characteristics, including the Youden index, sensitivities, specificities, positive predictive values, and negative predictive values. The authors used the Youden index to identify optimal cut-offs for each model according to the outcome of interest.

The authors also compared the predictive performances from our models and the National Early Warning Score-2 (NEWS-2)^19^ and 4C Mortality Score.^20^ The authors used reclassification tables and measures of net reclassification improvement (the net percentage events correctly classified upward) and integrated discrimination improvement (difference in discrimination slopes between two models).

### Ethical aspects

The institutional ethics committee reviewed and approved our research protocol with an exemption of informed consent. The authors kept all identifiable patient information confidential throughout the study.

## Results

During our recruitment period, 3,596 patients (> 14 years) were admitted to HCFMUSP with suspected COVID-19. Of those, 574 candidates were excluded due to a lack of laboratory confirmation of SARS-CoV-2 infection. The final study sample included 3,022 participants.

The overall demographics and clinical characteristics of the study participants are presented in Table 1, according to hospital mortality. Compared with non-survivors, a lower percentage of survivors were male (52% vs. 62%, p < 0.001). Survivors were also younger (p < 0.001) and less likely to have diagnosed comorbidities, except for liver disease, HIV, and hematological cancer. Table 2 presents the baseline reported symptoms of study participants, overall and according to hospital mortality. The percentage of patients reporting good general health conditions was higher among survivors. Interestingly, flu-like symptoms were more frequently reported by survivors. Median SAPS-3 values were higher among non-survivors. Baseline laboratory findings are described in Table 3. Measurements of complete blood count, kidney function, liver enzymes, C-reactive protein, lactic dehydrogenase, creatine kinase, albumin, prothrombin time, and D-dimer were all consistently and significantly abnormal in non-survivors.

**Table 1:** Baseline demographic and clinical characteristics of study participants, according to hospital mortality.

	**All patients** **(n = 3,022)**	**Survivors** **(n = 1,968)**	**Non-survivors** **(n = 1,054)**	**p-value**
Mean age (SD)	59 (16)	56 (17)	66 (14)	< 0.0001
Median age (IQR)	61 (48–71)	57 (44–68)	68 (59–75)	< 0.0001
Age categories (%)				< 0.001
< 40 years old	426 (14)	376 (19)	50 (5)	
40‒49 years old	411 (14)	332 (17)	79 (8)	
50–59 years old	561 (19)	400 (20)	161 (15)	
60–69 years old	769 (25)	451 (23)	318 (30)	
70–79 years old	562 (19)	281 (14)	281 (27)	
80+ years old	293 (10)	128 (7)	165 (16)	
Male sex (%)	1,682 (56)	1,030 (52)	652 (62)	< 0.001
Race/skin color (%)^a^				0.872
White/Caucasian	1,877 (62)	1,223 (65)	654 (64)	
Black	223 (7)	142 (8)	81 (8)	
Mixed	786 (26)	502 (27)	284 (28)	
Asian	28 (1)	17 (1)	11 (1)	
Education level (%)^b^				< 0.001
Illiterate	75 (6)	33 (6)	42 (6)	
Elementary	316 (25)	93 (16)	223 (32)	
Middle	486 (38)	256 (43)	230 (33)	
High	251 (20)	121 (21)	130 (19)	
College/University	151 (12)	87 (15)	64 (9)	
Hypertension (%)^c^	1,735 (57)	1,059 (54)	676 (64)	< 0.001
Diabetes mellitus (%)^c^	1,119 (37)	668 (34)	451 (43)	< 0.001
Obesity (BMI ≥30) (%)^d^	744 (32)	540 (37)	204 (24)	< 0.001
Cardiovascular disease (%)^e^	561 (19)	342 (17)	219 (21)	0.023
Stroke (%)^f^	214 (7)	117 (6)	97 (9)	0.001
Dementia (%)^f^	100 (3)	43 (2)	57 (5)	< 0.001
COPD (%)^f^	188 (6)	101 (5)	87 (8)	0.001
Rheumatologic disease (%)^c^	75 (2)	57 (3)	18 (2)	0.045
Liver disease (%)^c^	90 (3)	51 (3)	39 (4)	0.088
Chronic kidney disease (%)^g^	274 (9)	147 (7)	127 (12)	< 0.001
HIV (%)^f^	30 (1)	19 (1)	11 (1)	0.837
Cancer (%)^h^	313 (11)	153 (9)	160 (15)	< 0.001
Hematological cancer (%)^i^	87 (4)	44 (3)	43 (5)	0.062

a Unknown for 108 participants (4%).

b Mssing for 1,742 participants (58%).

c Missing for 2 participants (< 1%).

d Missing for 930 participants (26%).

e Missing for 5 participants (< 1%).

f Missing por 1 participant (< 1%).

g Missing for 3 participants (< 1%).

h Missing for 231 participants (8%).

i Missing for 699 participants (23%). SD, Standard Deviation; BMI, Body Mass Index; COPD, Chronic Obstructive Pulmonary Disease.

**Table 2:** Baseline symptoms of study participants, according to hospital mortality.

	All patients (n = 3,022)	Survivors (n = 1,968)	Non-survivors (n = 1,054)	p-value
Days between the onset of symptoms and hospital admission				
Mean (SD)	9 (7)	9 (7)	8 (6)	0.155
Median (IQR)	8 (5–11)	8 (5–11)	7 (4–11)	0.060
General health condition (%)^a^				< 0.001
Good	1140 (52)	1028 (63)	112 (21)	
Regular	772 (36)	521 (32)	251 (47)	
Poor	261 (12)	89 (5)	172 (32)	
Fever (%)^b^	1,642 (56)	1160 (62)	482 (47)	< 0.001
Chills (%)^c^	153 (6)	120 (8)	33 (3)	< 0.001
Runny nose (%)^d^	309 (12)	225 (14)	84 (9)	< 0.001
Odynophagia (%)^e^	200 (7)	155 (9)	45 (4)	< 0.001
Myalgia/arthralgia (%)^f^	889 (31)	662 (36)	227 (22)	< 0.001
Dyspnea (%)^g^	2,224 (76)	1419 (75)	805 (78)	0.192
Cough (%)^h^	2,020 (69)	1353 (72)	667 (65)	< 0.001
Sputum (%)^i^	148 (8)	104 (9)	44 (7)	0.228
Loss of taste (%)^j^	403 (14)	333 (18)	70 (7)	< 0.001
Loss of smell (%)^k^	413 (15)	330 (18)	83 (8)	< 0.001
Loss of taste or smell (%)^l^	545 (19)	440 (24)	105 (10)	< 0.001
Headache (%)^m^	559 (20)	452 (25)	107 (10)	<0.001
Altered mental status (%)^n^	161 (6)	84 (6)	77 (8)	0.024
Nausea (%)^o^	307 (11)	236 (13)	71 (7)	< 0.001
Vomit (%)^p^	135 (7)	104 (8)	31 (5)	0.004
Abdominal pain (%)^q^	112 (4)	82 (5)	30 (3)	0.007
Diarrhea (%)^r^	370 (13)	275 (15)	95 (9)	< 0.001
SAPS-3^s^				
Mean (SD)	65 (17)	58 (14)	72 (16)	< 0.0001
Median (IQR)	65 (53–77)	58 (47–68)	72 (61–83)	< 0.0001

a Missing for 849 participants (28%).

b Missing for 109 (4%).

c Missing for 501 participants (17%).

d Missing for 478 participants (16%).

e Missing for 198 participants (7%).

f Missing for 162 participants (5%).

g Missing for 105 participants (3%).

h Missing for 114 participants (4%).

i Missing for 1,2011 participants (40%).

j Missing for 186 participants (6%).

k Missing for 176 participants (6%).

l Missing for 181 participants (6%).

m Missing for 499 participants (17%).

n Missing for 191 participants (6%).

o Missing for 1,027 participants (34%).

p Missing for 500 participants (17%).

q Missing for 178 participants (6%).

r Obtanied only for patients admitted to the ICU, thereby missing for 1,170 participants (39%).

s SAPS-3, Simplified Acute Physiology Score 3.

**Table 3:** Baseline laboratory findings of study participants, according to hospital mortality.

	All patients (n = 3,022)	Survivors (n = 1,968)	Non-survivors (n = 1,054)	p-value
Mean hemoglobin (SD)^a^	11.9 (2.4)	12.1 (2.1)	11.6 (2.6)	<0.0001
Mean leucocyte count (SD)^a^^,^*	9,903 (7,334)	9,096 (7,271)	11,423 (7,213)	<0.0001
Mean neutrophils (SD)^a^^,^*	8,125 (5,074)	7,168 (4,355)	9,944 (5,798)	<0.0001
Mean lymphocyte count (SD)^a^^,^*	1,148 (4,191)	1,308 (5,138)	844 (820)	0.0043
Mean neutrophil/lymphocyte ratio (SD)^a^^,^*	11.7 (13.0)	8.9 (9.1)	17.1 (16.9)	<0.0001
Mean platelets (SD)^a^	237,078 (108,917)	248,768 (109,697)	215,026 (103,963)	<0.0001
Platelets categories				<0.001
< 150,000 (%)	603 (20)	323 (17)	280 (27)	
150,000‒400,000 (%)	2,123 (72)	1,433 (74)	690 (68)	
> 400,000 (%)	221 (8)	170 (9)	51 (5)	
Mean urea (SD)^a^	64 (54)	51 (44)	88 (62)	<0.0001
Mean creatinine (SD)^a^	1.7 (2.1)	1.4 (2.0)	2.2 (2.1)	<0.0001
Mean C reactive protein (SD)^b^	150 (113)	128 (103)	193 (120)	<0.0001
Mean lactic dehydrogenase (SD)^c^	487 (384)	410 (242)	636 (536)	<0.0001
Mean creatine kinase (SD)^d^	680 (2594)	516 (2716)	963 (2341)	0.0007
Mean albumin (SD)^e^	3.0 (0.6)	3.1 (0.5)	2.8 (0.5)	<0.0001
Mean AST (SD)^f^	79 (363)	53 (94)	127 (595)	<0.0001
Mean ALT (SD)^f^	62 (198)	51 (98)	81 (304)	0.0002
Mean D dimer (SD)^g^	7,561 (19,590)	5,018 (15,904)	12,594 (24,599)	<0.0001
Mean prothrombin time (SD)^h^	14.0 (6.9)	13.8 (6.9)	14.4 (7.0)	0.0359

a Missing for 73 participants (2%).

b Missing for 294 participants (10%).

c Missing for 786 participants (26%).

d Missing for 1,362 participants (45%).

e Missing for 1,638 participants (54%).

f Missing for 482 participants (16%).

g Missing for 544 participants (18%).

h Missing for 640 participants (21%).

⁎ Excludes one participant with outlier leucocytes count who probably had hematological cancer.

From the complete cohort of 3,022 participants, 1,496 were randomly assigned to the derivation sample and 1,526 to the validation sample. In the derivation sample, 1,496 (68%) admissions required intensive care, and 527 (35%) participants died in the hospital. In the validation sample, there were 989 (65%, p = 0.077) ICU admissions and 527 (35%, p = 0.690) deaths.

After multivariable analyses, the following items were selected to predict ICU admission and hospital mortality (Table 4): age; cancer; dementia; diabetes; rheumatic disease; anosmia or ageusia; dyspnea; fever; headache; sore throat; C-reactive protein; creatinine; hemoglobin; neutrophil-to-lymphocyte ratio; platelets. All variables had a variation inflation factor of less than 1.5, indicating a lack of multicollinearity between predictors. Variables retained in the final models and their respective scores are presented in Table 4. The maximum scores, indicating the highest risk of ICU admission, were 48.5 points for Model 1 and 56.5 points for Model 2. The maximum scores, indicating the highest risk of death, were 29.0 points for Model 1 and 30.0 points for Model 2.

**Table 4:** Scores assigned to predictors in the final multivariable models for ICU admission and mortality.

		ICU-Model 1			ICU-Model 2			Death-Model 1			Death-Model 2	
Variables	Beta	95%CI	Score	Beta	95%CI	Score	Beta	95%CI	Score	Beta	95%CI	Score
Age < 50	1.06	(0.557–1.555)	+4.5	0.79	(0.376‒1.203)	+4.5	‒	‒	‒	‒	‒	‒
Age 50‒69	0.47	(0.092–0.847)	+2	‒	‒	‒	0.49	(0.099–0.876)	+1.5	0.40	(0.017‒0.783)	+1.5
Age ≥ 70	-	‒	‒	‒	‒	‒	0.86	(0.451‒1.269)	+3	0.82	(0.418‒1.225)	+3
Cancer = No	1.02	(0.514‒1.533)	+4	0.99	(0.519‒1.470)	+6	‒	‒	‒	‒	‒	‒
Cancer = Yes	‒	‒	‒	‒	‒	‒	0.67	(0.208‒1.130)	+2	0.60	(0.156‒1.045)	+2.5
Dementia = No	0.98	(0.213‒1.757)	+4	1.04	(0.301‒1.785)	+6	‒	‒	‒	‒	‒	‒
Diabetes = Yes	‒	‒	‒	0.35	(0.026‒0.666)	+2	‒	‒	‒	0.26	(-0.020‒0.532)	+1
Rheumatic disease = No	‒	‒	‒	‒	‒	‒	1.20	(0.142‒2.262)	+4	1.23	(0.214‒2.251)	+4.5
Anosmia or ageusia = No	0.38	(-0.035‒0.791)	+1.5	‒	‒	‒	0.58	(0.178‒0.976)	+2	‒	‒	‒
Dyspnea = Yes	‒	‒	‒	‒	‒	‒	0.43	(0.095‒0.758)	+1.5	‒	‒	‒
Fever = No	0.46	(0.124‒0.800)	+2	‒	‒	‒	0.30	(0.028‒0.582)	+1	‒	‒	‒
Headache = No	0.50	(0.078‒0.922)	+2	‒	‒	‒	‒	‒	‒	‒	‒	‒
Sore throat = No	0.51	(-0.084‒1.108)	+2	‒	‒	‒	‒	‒	‒	‒	‒	‒
Creatinine = 1.0‒1.4	0.48	(0.077‒0.876)	+2	0.60	(0.223‒0.977)	+3.5	0.42	(0.065‒0.780)	+1.5	0.49	(0.139‒0.837)	+2
Creatinine ≥ 1.5	0.82	(0.405‒1.228)	+3.5	0.90	(0.517‒1.292)	+5.5	0.97	(0.646‒1.300)	+3	1.01	(0.695‒1.335)	+4
Hemoglobin = 7‒8	0.24	(0.096‒0.385)	+1	0.17	(0.037‒0.302)	+1	‒	‒	‒	‒	‒	‒
Hemoglobin = 9‒10			+2		(0.037‒0.302)	+2	‒	‒	‒	‒	‒	‒
Hemoglobin = 11‒12			+3		(0.037‒0.302)	+3	‒	‒	‒	‒	‒	‒
Hemoglobin = 13‒14			+4		(0.037‒0.302)	+4	‒	‒	‒	‒	‒	‒
Hemoglobin ≥ 15			+5		(0.037‒0.302)	+5	‒	‒	‒	‒	‒	‒
Platelets < 150,000	‒	‒	‒	‒	‒	‒	1.14	(0.469‒1.803)	+4	1.16	(0.512‒1.799)	+4.5
Platelets = 150,000‒400,000	‒	‒	‒	‒	‒	‒	0.58	(-0.032‒1.202)	+2	0.55	(-0.046‒1.143)	+2
Platelets > 400,000	0.82	(0.065‒1.568)	+3.5	0.73	(0.044‒1.410)	+4	‒	‒	‒	‒	‒	‒
Neutrophil/lymphocyte ratio = 6‒15	0.95	(0.610‒1.300)	+4	1.01	(0.689‒1.338)	+6	0.66	(0.311‒1.015)	+2	0.78	(0.440‒1.130)	+3
Neutrophil/lymphocyte ratio > 15	2.15	(1.551‒2.741)	+9	2.16	(1.619‒2.707)	+12.5	1.31	(0.902‒1.717)	+4.5	1.39	(0.988‒1.785)	+5.5
C-reactive protein = 100‒199	0.40	(0.035‒0.766)	+1.5	0.43	(0.087‒0.782)	+2.5	0.51	(0.162‒0.856)	+1.5	0.53	(0.187‒0.866)	+2
C-reactive protein ≥ 200	1.32	(0.884‒1.764)	+5.5	1.26	(0.854‒1.672)	+7.5	0.72	(0.386‒1.057)	+2.5	0.78	(0.452‒1.106)	+3
**Maximum score = worse**			**48.5**			**56.5**			**29**			**30**
**Optimum cut-off (Youden)**			**25.5**			**26.5**			**15.5**			**15**

Beta coefficients are expressed in log-odds units. The beta coefficient for each retained variable was divided by the lowest beta coefficient in the model; the results were rounded to the nearest integer (0 or .5) to generate the respective score values in the new scoring systems.

*Dashes indicate variable was not part of the respective model.

**Fig. 1:**
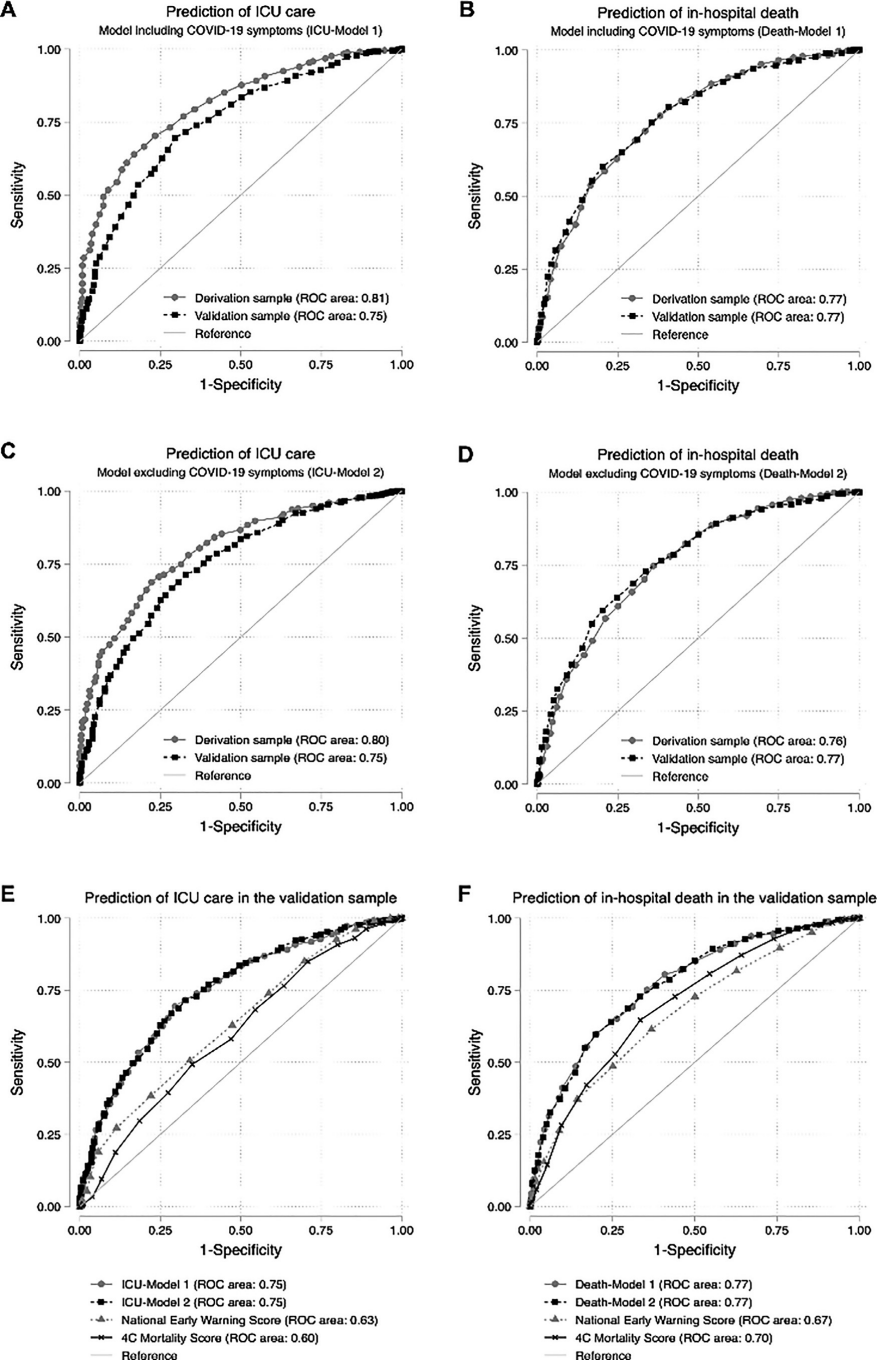
Receiver operating characteristic curves for the prediction of ICU admission and hospital death.

Fig. 1 presents ROC curves examining the accuracy of Models 1 and 2 in predicting ICU admission (Panels A and C) and hospital death (Panels B and D). The areas under the ROC curves were very similar for the derivation (grey lines) and validation samples (black lines). Both scores derived from Models 1 and 2 for ICU admission had a 75% (95% Confidence Intervals [95% CI] 72%‒78%) accuracy in the validation sample, whereas both scores derived from Models 1 and 2 for death had a 77% (95% CI 74%‒80%) accuracy in the validation sample, suggesting the inclusion of flu-like symptoms to the models did not remarkedly improve their discrimination capacity. Panels E and F contrast the accuracy of scores derived from Models 1 and 2 with the previously published NEWS-2 and 4C Mortality Score in the validation sample, demonstrating that our models were more accurate in predicting ICU admission and hospital mortality. For ICU admission, the areas under the ROC curves were 0.63 and 0.60 for NEWS-2 and 4C Mortality scores, respectively. The areas under the ROC curves for mortality were 0.67 and 0.70 for NEWS-2 and 4C Mortality scores, respectively.

## Discussion

In this study, the authors used a detailed dataset of patients admitted to a large academic COVID-19 treatment center in Brazil to identify factors associated with ICU admission and death. The authors built predictive scores that can be used in hospitals and emergency healthcare units to support clinical decisions and resource allocation. The final scores included age, comorbidities, and baseline laboratory data and were more accurate than the previously published NEWS-2 and 4C Mortality Score. Furthermore, the authors found that including baseline flu-like symptoms in the scores did not add substantial value to their accuracy.

Several studies including both outpatient and hospitalized participants have explored prognostic scores in COVID-19. A recently published systematic review^21^ examined articles published up to May 2021 and identified 79 studies investigating prediction models for severe COVID-19. Nevertheless, most had significant methodological caveats and were rated as having a high risk of bias or high concerns for applicability. Out of the nine studies rated with a low risk of bias and low concerns for applicability, one included patients with suspected COVID-19;^22^ one addressed respiratory failure as an outcome;^23^ one included variables collected one week after hospital admission;^24^ and three included COVID-19 patients in outpatient settings.^25^, ^26^, ^27^ The remaining three studies developed risk scores for mortality in hospitalized patients with laboratory-confirmed COVID-19. Some studies used chest roentgenogram and computed tomography findings as predictive variables either alone or with clinical data. In our study, patient radiology findings were unavailable and could not be included in the models. Even so, the authors had a detailed database of more than 3,000 individuals, and we were able to explore prediction models using 25 demographic, clinical, and laboratory variables.

Chen et al. developed the OURMAPCN-score using data from more than 6,000 patients admitted to seven hospitals in Wuhan as the derivation sample, with an external validation sample including more than 9,000 patients from China and Italy. The score included admission before the national maximum number of daily new cases was reached, age, oxygen saturation, blood urea nitrogen, respiratory rate, procalcitonin, C-reactive protein, and absolute neutrophil counts. Of note, this score included procalcitonin, a marker of systemic inflammation that is not readily available in most hospitals. Moreover, it included a calendar reference that is unlikely to apply to other settings. More recently, the same research group developed the PAWNN score, which used only age and complete blood count information (platelet counts; white blood cell counts; neutrophil counts; and neutrophil-to-lymphocyte ratio) as variables for a prediction tool built using a derivation sample of more than 9,000 patients and a validation sample of almost 3,000 patients in China; in this analysis, the model accuracy was 80% in an external validation sample including 227 patients from Italy.^28^

Knight et al. used data from the International Severe Acute Respiratory and Emerging Infections Coronavirus Clinical Characterisation Consortium (ISARIC-4C) to build the 4C Mortality Score. The study included more than 35,000 patients in the derivation sample and more than 22,000 patients in the validation sample. The final score included age, sex, number of comorbidities, respiratory rate, peripheral oxygen saturation, level of consciousness, urea, and C-reactive protein levels. In addition to the high discrimination for mortality, the 4C Mortality Score had the advantage of including variables usually available at the initial hospital assessment.^20^

In our prognostic score, we used variables readily available in most hospital settings that could be applied in different scenarios. We compared our scores with the 4C Mortality Score20] and the widely validated NEWS-2^19^ and observed that our discriminatory performance was higher. In a previous study, Bradley et al. showed that already-established prognostic scores may underestimate mortality in COVID-19 patients.^29^ Another study in our institution found a poor prediction performance of the original version of NEWS, qSOFA, and SIRS to predict mortality, early bacterial infection, and admission to ICU in COVID-19 patients admitted to the emergency department.^30^ Furthermore, our hospital participated in a binational study including 1,361 patients from Brazil and Spain to evaluate the performance of 11 risk stratification scores in predicting hospital mortality and ICU admission. The results of the study indicated that the more recent scores created to predict COVID-19 outcomes had a similar ability to predict mortality compared to the conventional pneumonia scores. However, all the scores demonstrated inadequate performance in predicting ICU admission.^31^ Together with our findings, these results highlight the need to recalibrate or develop specific prognostic scores in the context of different diseases and settings.

Despite the initial optimism brought on by the development of several effective vaccines for COVID-19, their generally slow rollout and the emergence of new SARS-CoV-2 variants have contributed to recurring waves of infected patients in several countries.^1^ Despite being less severe compared to the situation prior to the availability of vaccines, the persistent strain on emergency departments and hospitals highlight the ongoing need for efficient resource allocation. Moreover, the epidemiological data underlines the importance of regularly reassessing the factors that contribute to adverse outcomes in hospitalized COVID-19 patients. This should be done while keeping in mind the continuously evolving variables such as vaccination status, previous exposure to the virus, and therapeutic interventions such as antiviral medications and monoclonal antibodies.

This study had limitations. The authors used data from a relatively small sample (3,022 individuals) admitted to a single tertiary university hospital in a resourceful city in Brazil. However, our hospital was the primary referral center for severe COVID-19 in Sao Paulo, receiving patients from all regions of the metropolitan area, which is the most populated in Brazil with 23.5 million inhabitants. All participants were enrolled in 2020, preceding any exposure to previous SARS-CoV-2 infection or vaccination and prior to the emergence of viral genetic variants, which will likely modify the disease prognosis. As such, it is unlikely that our scores could be directly applied to contemporary cohorts of hospitalized COVID-19 patients. The authors also limited our analyses to patients aged ≥14 years and cannot extrapolate our results to pediatric populations. Nevertheless, our results are valuable in showing that prognostic scores created using readily accessible, locally sourced data and easily managed through electronic health records can be more effective in predicting clinical outcomes and improving resource allocation compared to scores developed externally.

## Conclusion

In conclusion, the SARS-CoV-2 pandemic has resulted in a massive public health crisis, putting significant strain on healthcare systems globally. This highlights the critical need to optimize resource utilization, particularly in the face of supply shortages. Prognostic scores, created using locally sourced and easily accessible information and validated on contemporary patient cohorts, are critical tools in supporting clinical decision-making and maximizing the impact of limited healthcare resources.

## Declarations

Ethics approval and consent to participate: The institutional ethics committee ‒ Comissão de Ética para Análise de Projetos de Pesquisa – CAPPesq ‒ reviewed and approved our research protocol (n° 31382620.0.0000.0068, 30417520.0.0000.0068, and 32037020.6.0000.0068) with an exemption of informed consent.

## Consent for publication

Not applicable.

## Availability of data and materials

The datasets used and/or analyzed during the current study are available from the corresponding author upon reasonable request. HCFMUSP will participate in the COVID Brazil Data-Sharing repository coordinated by The State of São Paulo Research Foundation (FAPESP), providing open access to hospital data related to COVID-19 hospitalizations (https://repositoriodatasharingfapesp.uspdigital.usp.br/).

## Authors' contributions

VAS, TAS, and EGK conceived the study. VAS and TAS performed data analysis and drafted the first version of the manuscript. TAS prepared Figure 1. MJRA and JCF contributed to the manuscript writing. JFM, VCJ, MMF, KRS, JEP, NMS, LA, AJSD, MMM, TEPBF, CC, and HPS contributed to data acquisition and database organization. All authors revised and approved the final version of the manuscript.

## Funding

The authors acknowledge the financial contribution to the study setup provided by donations from the general public under the HC-COMVIDA crowdfunding scheme (https://viralcure.org/c/hc) with funds managed by the Fundação Faculdade de Medicina. MJRA was supported by a scholarship from HCFMUSP with funds donated by NUBANK under the #HCCOMVIDA initiative.

## Conflicts of interest

The authors declare no conflicts of interest.
